# An Overview on T-Cell Engagers: The Current Position in Both the Biological and Mathematical Context

**DOI:** 10.34133/csbj.0209

**Published:** 2026-09-11

**Authors:** Jonathan Davies, Liam V. Brown, Mark W. Jones, Mark Penney, Gibin Powathil

**Affiliations:** ^1^ Department of Mathematics, Swansea University, Swansea, UK.; ^2^ Early Oncology DMPK, Oncology Targeted Discovery, AstraZeneca, Cambridge, UK.; ^3^ Department of Computer Science, Swansea University, Swansea, UK.

## Abstract

T-cell engagers (TCEs) are immunotherapeutic drugs that are gaining interest and momentum in oncology, and since 2022, they have been increasingly approved for clinical use. However, there are still many issues and unknowns relating to their pharmacokinetics, efficacy, and adverse side effects. Mathematical models are in the perfect position to improve understanding of these key issues, expand on, and optimize currently approved TCEs. This paper will review the relevant biology behind TCEs, such as the mechanisms behind the antitumor response, the molecular biology of TCE construction, and their use in clinical settings. Then, the review will move to discuss TCE mathematical modeling, key methods, and conclusions to date.

## Introduction

With the increased rate of approval of T-cell engagers (TCEs), the understanding and interest of their potential are accelerating likewise. However, being an immunotherapy, TCEs have many moving parts that range from TCE design, the immune system, and cancer dynamics, bringing unique challenges in optimizing their design and implementation. These interconnected mechanisms make both the biological and mathematical perspectives come with their own unique complexities. As the rate of TCE approval increases, so does the complexities of the respective fields, which may add difficulty to collaboration between the two. Therefore, a review that is approachable for both fields is invaluable to allow both fields to fully benefit from recent advancements. This review will discuss the biological mechanisms and dynamics of TCE, and then delve into the challenges these dynamics present to TCE that prevent them from reaching full potential. The review will then focus on the mathematical perspective of TCE, delving into the different strategies being used to model TCE and insights learned from it. The review will also discuss the unique perspectives we learn from TCE modeling that traditional *in vivo* and *in vitro* experiments cannot provide.

## The Mechanisms behind TCEs

### T cells

T-cell dynamics play a vital role in how TCEs induce death in cancer. The basic mechanisms and roles of T cells in the immune system are as follows. The immune system is made up of 2 systems, the innate and adaptive. The innate system is the oldest part, which responds rapidly to common threats by means of pattern recognition receptors that can detect bacteria or other non-self cells. However, the innate system may not always be able to resolve issues by itself and so will need to rely on help from the adaptive immune system. Unlike the innate system, the adaptive system is slow to respond and takes time before it builds up a substantial response. The adaptive immune system has the ability to recognize practically any organic molecule. A molecule or fragment of a molecule that is identified as non-self, an “antigen”, triggers the expansion of immune cells that are able to recognize and destroy molecules and cells that contain this antigen, allowing for a more precise and effective response than the innate immune system [1]. T cells compose a substantial part of the adaptive system, responsible for destroying foreign or infected cells by cytolysis. Initially, a T cell will be naive, meaning T cells are less effective at causing cytolysis. Naive T cells traffic repeatedly between the blood and secondary lymphoid organs (primarily the lymph nodes and spleen) until they are activated via presentation of an antigen by antigen-presenting cells such as dendritic cells [2]. Dendritic cells are cells positioned all over the body, which collect and process antigen from tissues for presentation to cells of the adaptive immune system. Once a dendritic cell has collected antigen, it begins to traffic to secondary lymph tissue, primarily lymph nodes. Activation of the dendritic cell by local cytokines may expedite this process. The dendritic cell presents collected antigen on its cell surface within structures known as major histocompatibility complex (MHC) molecules, which can be recognized by cells of the adaptive immune system [1,3].

Each T cell that matures from the thymus expresses unique T-cell receptors (TCRs) that each have an affinity to different organic molecules. For each given antigen, roughly 1 in 10^4^ to 1 in 10^6^ T cells will recognize them. Antigen-presenting cells also provide costimulatory signals to the T cells that promote activation and maturation of naive T cells into effector or memory cells. T cells that are activated by means other than binding to antigen-presenting cells may be shorter-lived and less cytotoxic [1,4]. An antigen that is recognized by a given T cell is said to be “cognate” to it [5–8]. Once a T cell is activated, it will travel to the location of the immune response in order to fulfill its role in the immune response [2]. Generally, MHC-I molecules are designed to present antigen to cytotoxic T cells and MHC-II molecules are designed to present peptides to helper T cells. Helper T cells secrete cytokines to promote an immune response while regulatory T cells secrete cytokine to suppress an immune response [1]. These cells are vital for staging an immune response while preventing the immune system from overreacting. However, TCEs are generally designed to improve the performance of cytotoxic CD8^+^ T cells. Most cells in the body present MHC I molecules, which present peptides taken from the inside of their cell. Cytotoxic CD8^+^ T cells bind to MHC-I molecules and, if they recognize their cognate antigen within the MHC-I molecule, it will form a stronger immunological synapse with the target cell. This synapse usually results in the target cell’s death by apoptosis.

### T-cell engager

**Fig. 1.**
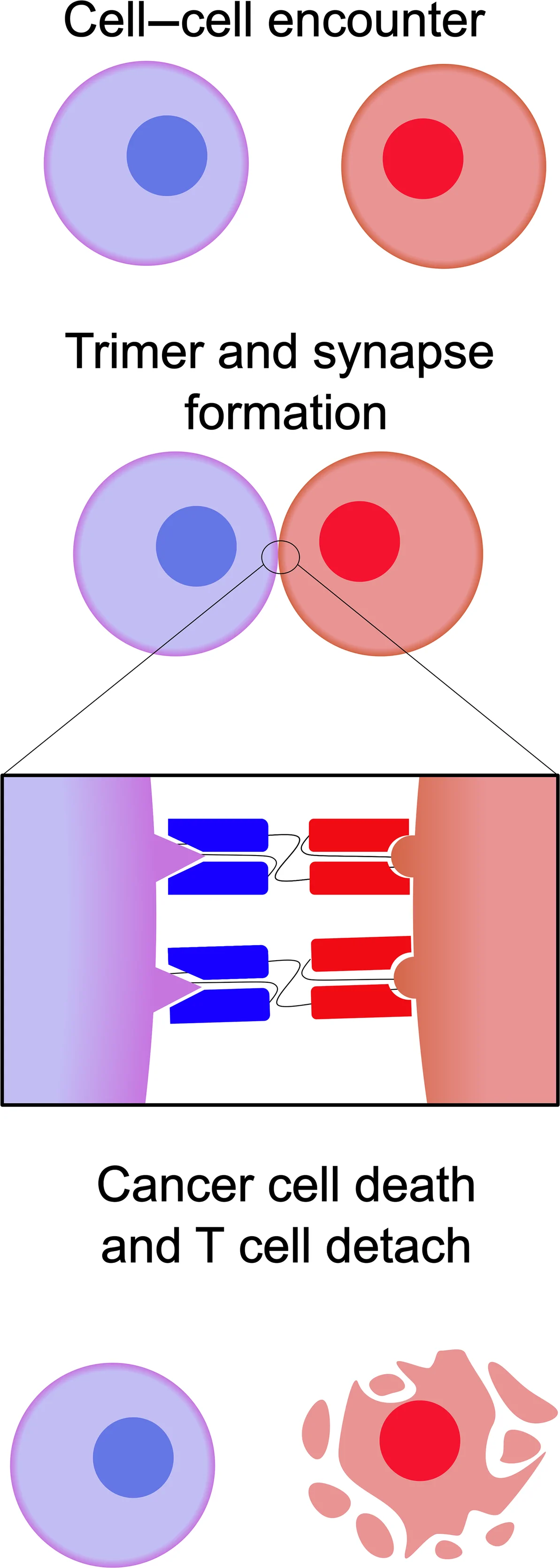
An illustration showing how TCEs facilitate cell killing. When a T cell and cancer cell encounter their corresponding dimers attach to the opposite cell’s receptors to create trimers. If enough trimers are formed, the T cell creates a synapse with the cancer cell, leading to the cancer cell’s death.

TCEs are drugs that bind to a T-cell antigen (commonly CD3) and to a tumor-associated antigen (TAA). By binding to both the T cell and the target cell, the TCE creates a synapse leading to the death of the target cell. This allows for T cells to be redirected toward target cells, causing increased killing of the target cells, especially in cases where the target cells would not normally be recognized by T cells, i.e., would not normally present non-self antigen. This synapse is MHC independent, meaning the T cell can be activated and kill the target cell without the need to go through the MHC scanning and activation mechanisms [9]. A diagram displaying how TCE binding works can be seen in Fig. 1. TCE binding between target cell and T cell can be used to treat cancer, by creating TCEs that bind to antigens that are either uniquely or overly expressed on cancer. Many of the currently approved TCEs are designed to treat cancer for both blood and solid cancers, and are typically administered intravenously or subcutaneously. Much of the clinical study design following administration concerns management of the side effect profile. However, both cancer and T cells have complex biological structures and dynamics, which creates further complexity when trying to predict the pharmacodynamics of TCEs with cancer.

### TCE: Classical designs and their characteristics

TCEs are developed using different techniques and with different structures. The difference in these structures can influence dynamics of the TCE such as half-life and tumor permeability, which in turn changes the pharmacokinetics and pharmacodynamics of the TCE in the body. Each design of TCEs comes with its own unique advantages and disadvantages. Generally, many TCEs can be split into 2 designs: immunoglobulin G-based antibodies (IgG-like) TCEs and variable fragment-based antibodies (Fv-like) TCEs [10]. While there is variation in these designs, they all share some commonality, such as a binding site for the T cell and a binding site for the TAA.

The majority of currently marketed TCEs are the IgG-like TCEs, and were the first TCE design developed [11,12]. These TCEs mimic the appearance of naturally occurring antibodies. These TCEs have 2 antigen binding regions on Fab arms, 1 that binds to T cells and 1 to the TAA. These antigen binding regions are connected to a hinge, which connects to a Fragment crystallizable (Fc) domain. This Fc domain is the biggest difference between IgG-like TCEs and other TCEs. The Fc domain has the capability to bind to other cells and stimulate immune cells like natural killer cells and macrophages [10]. However, this can contribute to cytokine release syndrome (CRS), off-target toxicities, and, in some cases, reduced efficacy [13]. To address the contribution to the adverse side effects, interaction of the Fc with Fcgamma receptors is mutated out to limit the risk of adverse side effects caused by Fc binding while keeping the structural benefits. The structural benefit of the IgG-like TCE is that the larger size of the TCE increases the molecular weight to one similar to an IgG, preventing rapid elimination via glomerular filtration and further extending the molecule’s half-life by permitting FcRn-mediated recycling, causing similar pharmacokinetics to IgG antibodies [13,14]. Consequently, this can allow for less intensive dosage regimens, compared to TCE with shorter half-lives. The basic format of the IgG-like TCE can be extended with further Fab or VHH binding arms at the C or N termini, to engage additional T cell (e.g., CD28) and TAA targets.

After the first designs of IgG-like TCE were created and studied, a new design for antibody-like molecules was developed, called the single-chain variable fragment (scFv) [15,16]. Soon after scFv development, research went into developing a TCE without the Fc receptor [17], which are now commonly referred to as Fv-like TCEs. These TCEs consist purely of light chains and heavy chains, which bind to TAA and T-cell antigens. With the smaller molecular weight compared to IgG-like TCEs, Fv-like has a high tumor tissue permeability, but a smaller half-life [10,13]. The lack of FC domain also allows for greater tissue distribution and access to the cancerous cells [18]. The structure of FV-like TCE leads to easier to manufacturing and lower immunogenicity [10,19]

When designing a TCE, the choice of linkers can potentially change the properties of the TCE. Short chains can prevent intra-linking of the heavy and light chains of the binding regions, while longer chains can improve TCE flexibility, allowing for easier antigen binding. More chains can be added to improve the TCE stability at the cost of flexibility [20].

While many TCEs may be categorized into an IgG-like TCE or Fv-like TCE, these classical categories are too rigid to describe the structures of newer TCE that are being developed. Tarlatamab is an example of a TCE that does not fit into either of the classical categories. The Tarlatamab structure consists of an anti-DLL3 bispecific TCE fused to an Fc domain [21]. Without the Fc domain, Tarlatamab would fall into the category of an Fv-like TCE; however, the shape of Tarlatamab does not mimic an IgG, which makes the classification of IgG-like also inadequate as a descriptor. Taralatamab and similar TCEs are often classified as half-life extended TCEs [10,21]. Half-life extended TCEs are designed in order to allow properties from both of the classical categories such as the half-life of an IgG-like TCE, but the flexibility of an Fv-like TCE.

#### Half-lives

To help visualize the difference between the different half-lives of categories of TCEs, Table 1 lists the type and estimated half-lives of various approved and nonapproved TCEs as reported. Different sources report the half-lives with different metrics. We cite the half-lives alongside reported definitions as applicable. In cases where human data were unable to be found, primate and mice data were instead reported. While differences in biology mean that nonhuman half-lives cannot directly translate to human half-lives, it is still useful to highlight trends of half-lives. As seen in the table, Fv-likes are generally limited to a time frame of hours, while half-lives of extended and IgG-like TCE generally have a time frame of weeks. This becomes an important consideration when deciding correct dosing and the potential pharmacodynamic and pharmacokinetic each TCE may have.

**Table 1.** The structure and half-lives of differing TCE

TCE half-lives					
TCE name	Structure	Target	Reported half-life	Species	Source
Blinatumomab	Fv-like	CD3 × CD19 B-cell acute lymphoblastic leukemia	2.11 h (elimination)	Human	[162]
AMG 211	Fv-like	CEA × CD3 gastrointestinal adenocarcinomas	3.3 h (tracer serum)	Human	[163,164]
Pasotuxizumab (AMG 212)	Fv-like	PMSA × CD3 castration-resistant prostate cancer	7 h subcutaneous formulation, 1–3 h intravenous administration (serum)	Human	[165,166]
Solitomab (MT110, AMG 110)	Fv-like	EpCAM × CD3 solid tumors (such as colorectal, ovarian, and gastric)	4.5 h (elimination)	Human	[167]
Flotetuzumab (MGD006,S80880)	Fv-like	CD123 × CD3 refractory acute myeloid leukemia (AML) and myelodysplastic syndrome (MDS)	3.5 h (elimination and terminal)	Cynomolgus monkey	[168,169]
AMV564	Fv-like	CD33 × CD3 AML,MDS and melanoma	2–3 days (terminal)	Human	[170,171]
AMG330	Fv-like	CD33 × CD3 AML	6 h (serum)	Nonhuman primates	[172,173]
Half-life extended (HLE) AMG 330	Fv-like half-life extended	CD33 × CD3 AML	44–167 h (serum)	Nonhuman primates	[172,173]
Tebentafusp	Nonclassical	gp100 × CD3 unresectable or metastatic uveal melanoma	6–8 h (terminal)	Human	[174,175]
Tarlatamab	Fv-like half-life extended	DLL3 × CD3 small cell lung cancer	11.2 days (terminal)	Human	[21,176]
PF-06671008	Fv-like half-life extended	P-cadherin × CD3 solid tumors overexpressing P-cadherin (e.g., gastric, pancreatic, and colon)	4.4 days (terminal)	Knock-in mice	[177,178]
MGD011	Fv-like half-life extended	CD19 × CD3 B-cell malignancies	14.3–20.6 days (terminal)	Humans	[179]
HPN217	IgG-like	BCMA × CD3 relapsed/refractory multiple myeloma	68 h	Human	[180]
Mosunetuzumab	IgG-like	CD20 × CD3 follicular lymphoma	16.1 days (terminal)	Human	[181,182]
Teclistamab	IgG-like	BCMA × CD3 relapsed or refractory multiple myeloma	27.2 days (elimination)	Human	[183]
Epcoritamab	IgG-like	CD20 × CD3 relapsed or refractory large B-cell lymphoma and relapsed or refractory follicular lymphoma	22 days (terminal)	Human	[161,184,185]
Glofitamab	IgG-like	CD20 × CD3 relapsed or refractory DLBCL not otherwise specified, DLBCL arising from follicular lymphoma, or primary mediastinal B-cell lymphoma	7.6 days (terminal)	Human	[72,186]
Elranatamab	IgG-like	BCMA × CD3 relapsed or refractory multiple myeloma	22 days	Human	[187,188]
Talquetamab	IgG-like	GPRC5D × CD3 relapsed or refractory multiple myeloma	8.4–12.2 days (terminal)	Human	[189,190]
Alnuctamab	IgG-like	BCMA × CD3 relapsed or refractory multiple myeloma	13.6 days	Human	[191,192]

## The Current Limiting Factors Facing TCEs

### Immune escape

In some cases, although a patient may initially respond positively toward TCE treatment, the patient may relapse and gain drug resistance [22]. This is most commonly associated with antigenic loss [23], where the antigens required for treatment are down-regulated, mutated, or absent in cancer [23]. This would naturally decrease the efficacy of the treatment, as without the antigens required for TCE binding, the TCEs are unable to create sufficient trimers, composed of the T cell, TCE, and target cell, to form an immunological synapse and lead to activation of the T cell. The loss of antigen is common between both hematological malignancies such as myeloma [24] and leukemia [25] and solid tumors such as glioblastoma [26].

While the effect of antigen escape is generally the same in TCE therapies, the mechanisms behind antigen loss can vary and be caused by many factors. One of these factors may be cells not presenting the antigen gain a competitive advantage. The cancerous cells presenting the antigen will be killed by the therapy, while the cells without the antigen will not. This gives a selection advantage to the cells not expressing the antigen. This causes expansion of the cells that are not expressing antigen, leading to the cancer becoming resistant to the therapy [22]. Leukemia treatment often sees this phenomenon through lineage switching. Lineage switching is where the leukemia will be initially classified as one lymphoid or myeloid subtype, then after relapse change to a different subtype (normally expressing different antigens with resistance to the original treatment) [25].

Down-regulation is another type of antigen escape where the cancer stops expressing the required antigen itself. This can be caused by changes in cancer cell biology; an example of this was found in a blinatumomab study [27]. In this study, it was found that the cancerous B cells did not have reduced CD19 expression (the binding site of the TCE), but rather, CD19 was being expressed intracellularly rather than on the membrane. It was found that reduced CD81 expression was the cause, which is critical for CD19 maturation and trafficking to the membrane surface [22,27]. Another form of antigen escape is caused by the splicing of the target antigen, preventing binding to the TCE. In another blinatumomab study, it was found that splicing of the CD19 prevented blinatumomab from binding and was considered to cause antigen escape [22,28]. A reduction in target antigen may also occur through mutations of the target antigen themselves, meaning the antigen itself mutates in such a way the TCE can no longer bind to the antigen [29]. In a study on blinatumomab, it was found that mutations of CD19 in tumor cells lead to relapse of the patients, in which evidence suggested that in these relapsed patients, nearly all of their cancerous cells had undergone CD19 mutation [28]. The study found no evidence of these mutations before the treatments and argues that the mutation occurred during, and as consequence of, blinatumomab treatment. Target antigen loss through mutation is not unique to blinatumomab or CD19, as mutations of target cells have also been reported to be found in patients relapsing after other TCE treatments [24,30].

Mutations and changes in the cancerous cell may not be the only reason for antigen escape; it has been found that monocytes and macrophages can remove antigen from cancerous cells [25]. In a progress called trogocytosis, an Fc*γ* receptor-expressing immune cell forms a synapse with a cancerous cell. The immune cell will recognize the ligands on the cell, and will take up and internalize some cell membrane fragments and antigen [25,31]. Although direct evidence in TCE therapy remains limited, this mechanism is hypothesized to contribute to antigen loss during treatment. [32]. Furthermore, similar therapies like monoclonal antibodies [33] and chimeric antigen receptor (CAR) T cells [34] may share valuable insights into how trogocytosis may effect TCE.

### CRS and other adverse side effects

TCE treatment can lead to adverse side effects that complicate treatment and present a substantial risk to patients. These adverse side effects can become life-threatening and require intensive treatment. In a phase 3 blinatumomab trial, it was found that 98.5% of patients suffered from an adverse event with 19.1% of the patients suffering a fatal adverse event [35]. From this trial alone, it is self-evident how dangerous TCEs can be if not managed properly. In a phase 1 to 2 teclistamab trial, all 165 patients suffered an adverse event. It is important to note that 119 of these patients suffered from some grade of CRS [36]. This response is a key example of how, out of all the adverse side effects in TCEs, CRS has gathered a unique focus.

In recent clinical trials, CRS still has a high rate of occurrence [37]. The cytokines released by the activation of T cells leads to the activation of more immune cells, which creates an inflammatory response and, if not controlled, can cause a cytokine storm [38,39]. In most cases in TCE, CRS is primarily driven by anti-CD3-mediated T-cell activation; however, as previously discussed, other factors such as Fc-mediated stimulation of NK cells or macrophages may also lead to CRS. The uncontrolled inflammatory response can lead to mild to life-threatening symptoms such as fever, coughing, tachypnea, liver failure, renal failure, and respiratory failure [40–42]. The relative commonness and potential severity of CRS in TCE therapies highlight the importance of considering and managing CRS in any clinical setting.

A common biomarker of CRS is interleukin-6 (IL-6), in which a high expression of IL-6 is consistently found to correlate to CRS [40]. IL-6 is mostly considered a pro-inflammation cytokine; however, it has been found to also have anti-inflammatory traits [43,44]. In low levels of IL-6, the cytokine is responsible for promoting an anti-inflammatory response, often called classic signaling. However, in increased IL-6 levels, the cytokine is instead responsible for promoting a pro-inflammatory response often called trans signaling [39,45]. As IL-6 is highly expressed in cases of CRS, it is likely that some of the symptoms of CRS are from a pro-inflammatory IL-6-mediated signaling cascade [39]. However, it is important to note that IL-6 is not the only cytokine with increased expression in CRS. Other cytokines such as IL-10 and interferon-γ are also found to have increased expression in patients suffering with CRS and are also thought to play a key role in CRS dynamics [42]. A practical problem with using IL-6 as a biomarker is the need for measuring IL-6 levels in a hospital setting, as the assay required is not commonly available in hospitals. An ideal solution would be a biomarker that correlates to IL-6 levels, which can also be measured using pre-existing hospital equipment. C-reactive protein (CRP) is a phase reactant protein that is produced by the liver, and its levels increase in response to IL-6 levels [39,46]. A useful property of CRP is the lack of its diurnal and eating variations. Furthermore, CRP levels have very little interactions with drugs, making them a reliable measure of inflammation in the clinical setting [47]. CRP monitoring is already readily available in hospitals and has been shown to have some predictive ability of CRS [39,48].

While CRS is a large focus of the scientific community when it comes to adverse side effects of TCEs, it is not their only potential side effect. According to a study by Sayed *et al.* [49], around 20% of adverse effects are cardiovascular in nature, which can include bleeding, thromboembolism, and heart failure. Of those adverse events that are cardiovascular, around 30% resulted in mortality, whereas noncardiovascular adverse side effects have a 17% mortality rate [49]. The exact mechanisms behind TCE-related cardiovascular toxicities are not well defined. One hypothesis is that the damage is indirect; on-tumor activity raises the level of pro-inflammatory cytokines that subsequently lead to cardiotoxicity. Another is on-target, off-tumor activity, where TCEs cause T cells to engage with antigens shared between the tumor and cardiovascular tissue, leading directly to cardiotoxicity [50]. Neurological toxicities can also occur as a result of TCE therapies. Neurological toxicities often coincide with CRS; however CRS is not a necessity for neurological toxicities [36,37]. Mild symptoms can include headaches and tremors, where, in a study of blinatumomab, 38% of the neurological toxicities were headaches and 17% were tremors [51]. More severe symptoms include speech problems, neuropathy, and subdural hematoma, which, in the same study of blinatumomab, were found to occur with frequencies of 2.4%, 5%, and 1% to 2% respectively. These symptoms also occur in other TCEs, and other TCEs may also incur neurological toxicities such as hallucinations and seizures [37]. Neurotoxicity pathophysiology is similar to CRS and so many of the risk factors associated with CRS also applies to neurotoxicity [40]. It has also been proposed that for blinatumomab, T-cell adhesion to endothelial cells is required for neurotoxic effects [52]. This suggests that understanding and potential manipulation of T cells to prevent T cell to endothelial cell adhesion may reduce or prevent neurotoxicity. It is important to note that the toxicities discussed are not an exhaustive list of all toxicities present in TCE; other toxicities do exist and may need further research in order to understand their potential impact on patients, such as in the case of interstitial lung disease [53,54].

### Reduction of T-cell effectiveness over time

Any immunotherapy that uses T cells for cytotoxic killing will come across the issue of T cells losing effectiveness and the ability to induce a cytotoxic effect over time. One of the mechanisms in which this can occur is known as T-cell exhaustion [55]. T-cell exhaustion is the loss of effectiveness T cells acquire after prolonged interaction with antigen [56], a mechanism that regulates T cells to prevent overreaction to an infection. T-cell exhaustion is often a limiting factor for the longevity of immunotherapies that make use of T-cell cytotoxicity. T-cell exhaustion is expressed in differing forms, with some having greater interactions with cancer than others.

Inhibitory receptors play a large role in contributing toward T-cell exhaustion. As a T cell kills cells, it starts expressing more and more inhibitory receptors. These receptors, when engaged by the appropriate ligand through receptor-ligand binding, can inhibit T-cell activation and cause anergy or even cell death by apoptosis [57]. These receptors include PD-1, LAG-3, 2B4, and CD160 [58]. Out of these receptors, the inhibitory receptor that has garnered most interest, PD-1, is a major focus of the scientific community. PD-1 is a receptor that becomes more expressed the more exhausted a T cell becomes [59]. It binds to PD-L1 (sometimes referred to as CD274 or B7-H1) and PD-L2 (sometimes referred to as CD273 or B7-DC) [60]. The binding between PD-1 and its ligands leads to the inhibitory effects previously described. The reason for the great interest in PD-1 is the up-regulation of the ligands that bind to PD-1 in the tumor microenvironment, leading to greater levels of T-cell exhaustion [61]. There is evidence to suggest that the higher expression of PD-1 ligands has a material effect on T-cell exhaustion [62]. Therapies like immune checkpoint inhibitors that target these ligands have shown positive effects such as increasing survival rates and tumor regression [63]. With T cells expressing more PD-1 as they kill tumor cells and PD-L1 being up-regulated in the tumor microenvironment, the rate of T-cell exhaustion from being in the tumor microenvironment is massively increased.

While PD-1 is an important factor of T-cell exhaustion, other processes are also responsible for the loss of effectiveness from T cells. The immune system also regulates T cells that have been activated through a process called activation-induced cell death (AICD). This is a system the immune system uses to kill T cells through apoptosis, usually through Fas receptors and Fas-L ligands. Fas-L is a ligand found on T cells that binds to Fas for a cytotoxic effect [64]. However, T cells are not the only type of cell that expresses Fas-L. Research has found other cells, including cancerous cells, that also express the Fas-L ligand [65]. For AICD to occur in this manner, a T-cell’s Fas receptor may interact with its own Fas-L receptor or one from another cell [66]. While expression of Fas-L increases on a T cell after activation, a higher expression of Fas-L does not necessarily correlate with increased AICD susceptibility of a cell [65]. However, it has been shown that a repeatedly activated T cell becomes susceptible to AICD. A naive T cell expresses higher levels of an inhibitor of Fas-indicated death called FLIP. As a T cell becomes more and more activated, it expresses lower amounts of this inhibitor, and changes in Fas receptor signaling cause a T cell to become more susceptible to AICD [67].

### Hook effect

**Fig. 2.**
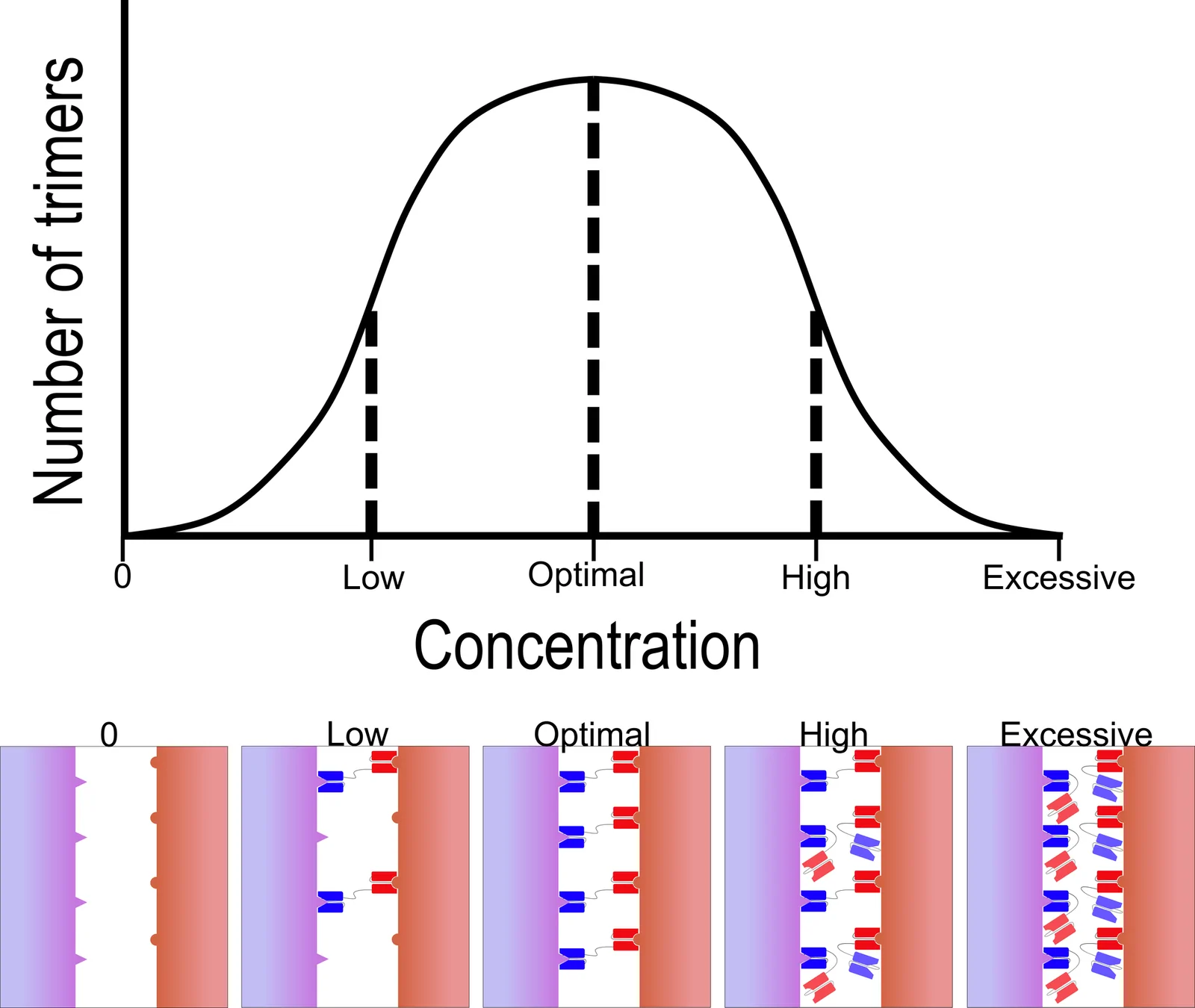
An illustration of the hook effect, showing how the number of trimer varies with TCE concentration. Each concentration level has an accompanying visualization of the cell walls, showing the trimer dynamics that occur between a T cell and cancer cell. Number of trimer increases with TCE concentration up until an optimal concentration; adding more TCE beyond this threshold causes the number of trimers to decrease as dimers begin saturating receptors, blocking the formation of trimers as the dimers have no free receptors to bind to.

As previously discussed, TCEs bind to the antigen on cells. However, cells have limited antigen for TCEs to bind to, creating competition between TCEs for the antigen. In theory, this could lead to scenarios in which the TCE creates tumor cell–TCE dimers and T cell–TCE dimers as expected. However, if there is too much engager compared to tumor antigen, then there is competition for antigen between free engager and T cell–TCE dimers, resulting in fewer trimers than would be expected. As TCEs require binding to both tumor cells and T cells for cytotoxic effect, this reduction in trimer concentration leads to reduced cytotoxicity. This is commonly referred to as the hook effect and has been observed in similar 3-body binding problems [68]. A graphical representation showing the typical curvature of the hook effect is shown in Fig. 2. Furthermore, *in vitro* experiments have shown the validity of this theory, where increasing TCE concentration past a maximum threshold leads to decreased tumor–T cell coupling efficiency [69]. Mathematical models of trimeric binding can reproduce the hook effect with both ordinary differential equation (ODE)-[70] and agent-based models [71]. It is important to note, however, that, as previously discussed, high concentrations of TCE have severe side effects such as CRS, meaning TCE concentration may not reach values where the hook effect is applicable to clinical settings [70]. As a result of the nature of clinical trials, the hook effect can be difficult to observe, which may cause uncertainty on its prevalence during treatment.

TCE concentration is not the only consideration for trimer formation. TCE design and antigen distribution on the cell may also play a factor in how effective a TCE is at forming trimers. Once a TCE has formed a dimer with one of the cells, the TCE is now limited by its flexibility and reach within a local radius of the initial binding site to form a second dimer and, hence, a trimer. This idea was modeled by Su *et al.* [71], in which they modeled different linker lengths on the TCE. In their findings, they suggest that there is an optimal length of the linker, where too short a linker reduces the area a TCE can bind to its second target, making trimers less likely to form. However, a longer linker also reduces trimer formation; Su *et al.* [71] speculated that too large a linker also results in fewer trimers being formed, speculating that the increased freedom of the TCE makes binding more difficult. The issue of antigen distribution becomes more relevant with TCE that bind to the same antigen on the same cell multiple times, such as glofitamab, which binds bivalently to CD20 on cancerous B cells [72]. The ability of the TCE to bind bivalently after the first bind will depend on the flexibility of the TCE and how much of the available antigen is distributed nearby. Hence, models that simulate bivalent binding to a singular cell, such as by Kaufman and Jain [73], may be applicable to TCEs. Their model restricts binding of the second antigen, depending on the radius the antibody can reach and the concentration of the antigen in that area. If the TCE engages 2 different antigens on the same cell, a model like the one reported by Rhoden *et al.* [74] may be more applicable. Rhoden *et al.*’s model [74] still incorporates the physical limitation of the number of antigen within reach of the initial binding site. However, Rhoden *et al.*’s model also includes competition between available antigen sites. Adaptation of these models to new or existing models to specialize them toward TCE dynamics could reveal new and novel results about the effect of bivalent binding to a singular cell on TCE efficacy and safety.

### Solid tumor characteristics

Many of the challenges facing TCEs are relevant to all cancer types. However, TCEs are currently showing more efficacy against hematologic malignancies than solid tumors, with the majority of the approved TCEs being for hematologic malignancies rather than solid tumors [75]. As the structure of solid tumors is uniquely different compared to hematologic malignancies, solid tumors propose unique challenges for TCE efficacy. An understanding of why TCEs are more effective against hematologic malignancies and the difficulties of solid tumors is an important concept to grasp in order to promote more effective development of TCEs for both cancers.

From the design stage of TCEs, solid tumors require more consideration when choosing which tumor antigen to bind to. Compared to hematologic malignancies, solid tumors have more heterogeneity, such as the type of antigens present on their cells [76]. This creates the issue of a lack of antigens that are completely unique to the cancer [77]. To an extent, this is also the case with hematologic malignancies; however, TCEs normally target antigens such as CD19 or CD20 when targeting hematologic malignancies. These antigens are normally shared only with other immune cells; thus, off-target toxicity will naturally mostly only affect immune cells, which are easily reproduced by the body [77]. When it comes to solid tumors, antigens chosen are not unique to the tumor but shared with the surrounding tissues and other organs, leading to on-target, off-tumor TCE binding to their cells. This can lead to organ damage from the therapy, which can come with its own severe complications (often referred to as on-target, off-tumor toxicity) [75]. On-target, off-tumor toxicity in solid tumors is further exacerbated by how solid tumors require higher exposure of the TCE for effective treatment [78], particularly as tissue concentrations of antibody-like drugs are usually lower than serum concentrations. The higher required dosage of TCE, and the abundance and availability of bindable antigen may be the reason CRS is generally more severe in solid tumors than it is in hematologic malignancies [79].

One of the biggest differences between solid and hematologic malignancies is the immunosuppressive tumor microenvironment. Solid tumors have a microenvironment where the cancer recruits immunosuppressive cells such as tumor-associated macrophages (TAMs) and regulatory T cells (Tregs). These immunosuppressive cells inhibit the killing potential of cytotoxic T cells, reducing the efficacy of TCEs [13]. Furthermore, the tumor microenvironment may also inhibit cytotoxic T cells through hypoxia, necrosis, nutrient deficiency, metabolic stress, immunosuppressive molecular factors (such as PD-1), and immune regulatory enzymes [77,80,81]. Depending on the activity of the cytotoxic T cell, the microenvironment can be categorized into 3 subgroups. The first is an inflamed tumor with cytotoxic cells infiltrating the tumor core, the second is an immune-excluded tumor that has only cytotoxic T-cell activity on the margin, and the third one is an immune desert tumor in which there is low or no cytotoxic T-cell activity [75,82,83]. Each microenvironment has unique properties that cause it to fall into its respective category. The inflamed tumor is the ideal case where cytotoxic T cells are engaging the tumor entirely. However, in the other 2 categories, the tumors express less immune activation biomarkers, which could lead to a lower immune response [75]. In the case of immune-excluded tumors, the T cells run into issues of penetrability because of physical and biochemical issues such as dense extracellular matrix and fibrotic tissue (physical) and a lack of chemotactic signaling for T cells (biochemical) [75,84]. Immune-excluded tumors may benefit from the increased permeability of Fv-like TCE, which could allow the T cells to infiltrate past the physical and biochemical barriers. Immune deserts share many of the properties of immune-excluded tumors, but generally will have less suitable antigens for T-cell activation and have a higher immunosuppressive microenvironment [75].

In many *in vitro* experiments, one of the biggest impact of efficacy on TCE is the T cell-to-tumor cell ratio (commonly referred to as effector cell:target cell ratio or E:T). It can be seen that the TCE may achieve much larger cell killing rate at higher T cell-to-tumor cell ratios [85,86]. These *in vitro* experiments will normally take place on a time scale of days, in contrast to the time scale of months *in vivo* treatments of TCE will take. These experiments will generally vary the E:T ratio between 10:1 and 1:10, and in the time frame allotted, it is seen that the high E:T ratios will achieve a high cytotoxicity, while the low E:T ratios will have a minimal cytotoxicity. As the T cells themselves are ultimately the ones responsible for cancer cell death in TCE therapy, it follows naturally that larger ratios of them result in quicker and higher cell kill. In hematological malignancies being treated with TCEs, inpatient studies have found that E:T can be 1:2 or 1:1 [87,88]. However, data on E:T ratios in solid tumors are much less readily available and more variable, and with solid tumors, this ratio is predicted to be much lower [89]. With a naturally lower E:T ratio compounding the microenvironmental issues previously discussed, this results in TCEs suffering from the slower and lower killing of target cells in solid tumors in a manner similar to the *in vitro* experiments.

## Proposed and Implemented Techniques to Overcome TCE Limiting Factors

### Step-up dosing

With CRS being such a prominent and serious side effect of TCEs, there is a substantial ongoing effort to reduce the occurrence and severity of CRS. It has been discovered that the incidence of CRS may be reduced by gradually activating a patient’s immune system. Instead of beginning treatment at the desired dosage, one starts at a lower dosage and slowly increases or “steps up” the dosage until the required dosage for the treatment is met. This principle was shown in a clinical trial for blinatumomab, where dosing at a third of the target dosage for a week and then stepping up to the target dosage resulted in less cytokine secretion compared to beginning at the target dosage for the treatment period [90,91]. Blinatumomab was approved by the Food and Drug Administration in 2014 [92], while the clinical trial for step-up dosage finished in 2016 [93]. This is indicative of the current trend in TCE approval where initially TCEs will be approved with standard non-step-up dosage treatments. Further studies and clinical trials will then evaluate the benefits of step-up dosage for the TCE, with future treatments of approved TCE implementing step-up dosage (with generally 1 to 3 escalations of dosage before reaching target dosage) [92].

The rationale behind step-up dosage is that CRS is most commonly observed around 4 to 16 h after the first dosage, and is normally confined to the first dosage of TCE [51,94]. Suggesting a lower initial dosage would make the CRS less severe. This would also result in the immune system having a more gradual increase of immune activity, causing future CRS occurrence and severity reduction. This is seen with common biomarkers associated with CRS like IL-6, where IL-6 levels did not increase beyond the initial peak from the original dosage of the TCE [90,95]. A possible factor is that tumor burden has been used as a predictor for severity of CRS, where a higher tumor burden correlates with more extreme CRS. As tumor burden is highest at the beginning of treatment, one would anticipate the most severe CRS then [41,42]. However, there is still more research required to fully comprehend the factors and influences behind CRS.

While step-up dosage is becoming increasingly more common for TCEs, deciding the amount and time scale of step-up dosage still remains a challenge. In some clinical trials, the step-up dosage was decided a priori, where the step-up dosage was taken as a “reasonable” fraction of the target dosage [90]. Considering the complex relationship between dosage, tumor burden, and other factors of treatment that can lead to CRS, a priori definitions of dosage levels are complex and could be aided with modeling. For some approved TCEs, the step-up dosage was decided using statistical and probabilistic models such as the continual reassessment method [96] and the Bayesian logistic regression method [92,97]. While these models help researchers decide the optimal dosage for TCEs, by their nature, they rely on observed data from clinical trials and are unable to predict dosages for new TCEs before trials. Development of mechanistic models that include key factors such as disease type, disease progress, and TCE trimer formation may assist with the calculation of optimal step-up dosage from preclinical data. This could also allow for new dosage regimens to be found and tested such as comparing normal step-up dosage to fractionated step-up dosage (in which the dosing interval is increased in the first cycle to provide similar overall exposure to a cycle at the target dosage [90]), while also giving new insights into the mechanisms of TCEs and CRS, which could help develop new strategies for dealing with the challenges of TCE.

### Intervention strategies

As previously discussed, TCEs come with many adverse side effects that require monitoring and treatment. These treatments may potentially have an impact on the efficacy of the immunotherapy [90]. Therefore an understanding of the general practice and treatment of adverse side effects is important to consider when developing TCEs.

As CRS is so common and severe in TCE therapy, much of the accompanying treatment is focused in reducing its incidence and severity. Treatment aimed at managing CRS can be preventive or reactive [94]. An example of a preventive strategy is to use intravenous fluid to reduce the risk of hypotension being caused by CRS [51,94]. While normally used in a reactive manner in order to treat symptoms, corticosteroids and antipyretics can be used preventively to reduce CRS symptoms [51,98]. In some TCE treatments, antimetabolites are used in the first few days of treatment as a preventive measure for CRS, with some research suggesting that hydroxyurea will not just reduce CRS severity but also increase target antigen expression on cancerous cells [98,99].

Reactive treatment of CRS depends on the grade of the CRS. At lower grades, the focus is on managing the symptoms of CRS rather than the CRS itself [39,94]. At higher grades, it is advised to not just focus on the symptoms of CRS, but to try and control and reduce the severity of the CRS itself. This may be done through temporary dose reduction or halting the TCE therapy. When trying to reduce and control the CRS, clinicians generally have 2 options: tocilizumab and corticosteroids. Tocilizumab is a monoclonal antibody that binds to and saturates IL-6 receptors, which inhibits IL-6 signaling [43]. Normally, one dosage is substantial enough to control CRS in most patients; however, a second dosage may be included if the first fails to control CRS in 6 to 8 h [41,94,100]. Tocilizumab also has application in the cases of concurrent neurotoxicity and CRS, as tocilizumab cannot cross the blood–brain barrier. This is useful in preventing problems that occur, inhibiting IL-6 signaling in the central nervous system [42]. Tocilizumab is generally the first drug used for CRS management as it is quick-acting and does not seem to negatively impact immune responses [38,42,101].

In some instances, corticosteroids can be used instead of or alongside tocilizumab. However, the use of corticosteroids may require more consideration in TCE therapy. Corticosteroids are synthetic hormones produced by the adrenal cortex and are a widely used drug for many different conditions outside of CRS and immune suppression such as reducing hypercalcemia, skin disorders, and hypoglycemia [102]. Corticosteroids diffuse through a cell membrane and binds to glucocorticoid receptors. The corticosteroid–glucocorticoid complex then diffuses into the cell nucleus and binds to glucocorticoid response elements [103]. These elements are associated with genes and result in the up-regulation of the transcription of anti-inflammatory genes and down-regulation of the transcription of inflammatory genes [104]. The transcription of the genes affects the production of pro-inflammatory cytokines, adhesion molecules, enzymes correlated to the immune system, and synthesis of immune cells, which are pro-inflammatory mediators (such as macrophages, mast cells, and dendritic cells) [102,103]. There is evidence to suggest that corticosteroids affect the production of B cells and T cells at high concentrations [102]. As corticosteroids affect more immune mechanisms than tocilizumab, there is controversy on whether the use of corticosteroids may affect the efficacy of immunotherapy. There is some evidence to suggest that corticosteroids affect T-cell persistence and expansion [48,105]. Alternatively, other studies into corticosteroids effects have suggested that corticosteroids have no effect on immunotherapy efficacy [106]. This has created a divide on the use of corticosteroids; some suggest using it as an alternative to tocilizumab or as premedication [39,94]. Alternatively, others suggest to use it as a second-line treatment for more severe CRS [39–41]. Corticosteroids do have the advantage of being able to cross the blood–brain barrier compared to tocilizumab, so in scenarios with neurological toxicities, corticosteroids are a more effective choice for treatment [42].

### Combination therapies

#### Immune checkpoint blockades

With TCE therapy being so reliant on the immune system, the use of immune checkpoint blockades as a combination therapy could lead to higher efficacy. Immune checkpoints are the pathways that regulate the immune system in order to maintain self-tolerance and reduce collateral tissue damage [107]. Examples of these checkpoints are PD-1 and CTLA-4, which inhibit T-cell cytotoxicity through the exhaustion mechanisms discussed previously in this review. Immune checkpoint blockades aim to block the inhibitory signaling of T cells and to reduce or, even in some cases, reverse the effects of T-cell exhaustion [108]. The blockading of these inhibitors can be achieved through antibodies that target TCRs or ligands [107]. By binding to the receptors or ligands, the normal receptor-ligand binding for inhibitory signaling is unable to be formed. However, the effects of immune checkpoint blockade can be limited by resistance to the therapy [109] or the activation of autoreactive T cells leading to organ specific autoimmune disease [108]. There is also emerging evidence to suggest that not all exhausted T cells may benefit from immune checkpoint blockade. Some exhausted T cells, referred to as progenitor exhausted T cells, express intermediate levels of PD-1 and display reduced cytotoxic function. Despite this, they maintain the capacity for self-renewal and can differentiate into other exhausted T-cell lineages [110,111]. Progenitor T cells are responsive to immune checkpoint blockade therapy, regaining proliferative ability and differentiating into T cells with full cytotoxic ability [112,113]. Alternatively, terminally exhausted T cells characterized by a loss of ability to proliferate, an incapacity to produce a cytotoxic effect, and a high coinhibitory receptor expression [112–114] lack responsiveness to immune checkpoint blockade therapy [112,113].

The use of immune checkpoint blockade and TCE as a combination therapy shows much potential from the theoretical point of view. T-cell exhaustion is a limiting factor of TCE therapy, which is supported by observations that blinatumomab treatment increases expression of checkpoints like PD-L1 [115–117]. The use of immune blockades to limit the effects of T-cell exhaustion could allow for greater efficacy. A combination strategy could be particularly beneficial in the treatment of solid tumors, which have a higher expression of immune checkpoints, as previously discussed. A quantitative systems pharmacology model developed by Ma *et al.* [118] explores this concept in different types of colorectal cancer in patients. In cases where the model predicts that monotherapy would not be effective, individual patients were found to benefit from combination therapy, which depended on patient-specific parameters. It was predicted that patients who transitioned from progressive disease to partial response/complete clearance, and progressive disease to stable disease during monotherapy may benefit from combination therapy. The promising *in silico* results have been replicated *in vivo* and *in vitro*, where immune blockade–TCE combination therapy resulted in increased efficacy against solid tumors [119,120]. Preclinical studies have also shown the complexity of the mechanisms behind the combination therapy. In a study involving a TCE and a CTLA-4 blocker, it was hypothesized that the increased antitumor efficacy may have resulted from the inhibition of Tregs [119]. However, other studies have shown that CTLA-4 blockers increase Treg proliferation [121]. The success of combination therapies may depend on the disease a given patient presents with. An *in vitro* study of a TCE–anti-PD1 combination therapy for T-cell acute lymphoblastic leukemia found that markers for T-cell exhaustion decreased, without any noticeable increase in cytolysis of target cells [122]. This indicates that multiple factors may be required for combination therapies to become advantageous over the monotherapy. Furthermore, the type of exhausted T cells of the patient should also be considered when considering if combination therapy will be effective. However, immune checkpoint–TCE combination therapies have already shown promise in clinical results [115].

#### CAR T cells

A class of immunotherapies closely linked to TCEs includes T-cell modifying therapies such as TCR T cells and CAR T cells. CARs are synthetic receptors that can be engineered onto T cells and other lymphocytes [123]. CAR T cells are produced using the patient’s own T cells. The patient’s T cells are firstly extracted from the patient, modified into CAR T cells, and then cultured and expanded to high enough numbers to have a clinical effect when infused back into the patient [124]. The mechanism of action of CAR T cells shares similarities with TCEs. The CAR binds to a specific antigen on a malignant cell to activate the lymphocyte and cause a nonclassical immune synapse to form, which leads to the CAR T cell killing the target cancer without the need for an MHC-I complex [125,126]. CAR T cells also share similar limitations and adverse events to TCEs, such as antigen escape, on-target, off-tumor toxicity, immunosuppressive tumor microenvironment, and CRS [123]. The management of these may require similar interventions to TCEs, such as tocilizumab and corticosteroids for CRS [127].

As CAR T cells and TCEs share many similarities, a combination therapy has the potential for synergetic effects. The proposed idea behind the synergy is that the TCEs can bind to the CAR T cells and tumor cells and promote a cytotoxic effect [128]. As previously discussed, antigen escape is a limiting factor of both CAR T cells and TCEs. However, this can be mitigated through the TCE and CAR T-cell targeting different TAAs. This concept has been proven in clinical trials. In one trial, a patient was treated with both a CD19 TCE and CD22 CAR T cell. The case found that when the CD22 CAR T-cell therapy lost effectiveness and the patient was relapsing, partially caused by a decrease in CD22 expression, introducing the TCE as a combination therapy increased cytotoxicity and promoted CAR T-cell proliferation [129,130]. This result is further supported by other studies where cells that had grown resistant to the CAR T-cell monotherapy were able to be targeted and killed by the introduction of TCE as a combination therapy [131]. In addition to combining them as 2 separate treatments, CAR T cells are being developed with the ability to secrete TCEs during the course of the therapy [132]. Clinical studies have found that secreted TCE from CAR T cells are better at redirecting bystander T cells than CAR T cell–TCE combination therapies [132,133]. Furthermore, in conditions of reduced antigen expression, secreted TCEs were found to exhibit a more potent cytotoxic effect with a faster onset than CAR T cell–TCE combination therapy [132]. Adverse side effects are a limiting factor in both therapies and so when applied as a combination therapy should be closely monitored. While management of these toxicities may be similar to monotherapy, the increased potency and efficacy of the treatment may lead to higher toxicities and adverse effects. However, some studies suggest that combination therapy may in fact reduce toxicities and allow targeting of antigens that would lead to too high a risk of off-target toxicities to be targeted with a monotherapy [133].

### Alternative TCE designs

In order to overcome the limiting factors of TCEs, new designs of TCEs are being developed. These new designs are generally aimed at either increasing therapeutic efficacy of the treatment or decreasing toxicities and adverse side effects. The new designs range from modifications to existing designs to entirely new designs outside of the IgG-like and Fv-like classifications.

Modifications to IgG-like TCEs and Fv-like TCEs may allow for more fine-tuning of their properties, without the need to completely redesign and develop new TCE. One potential modification is to create TCEs with binding sites to human serum albumin (HSA). Binding to HSA increases the molecular size of the TCE while also encouraging recycling of the molecules by the body, increasing the TCE half-life in the body [20]. Other methods to increase half-lives are available such as increasing the hydrodynamic volume by adding polyethylene glycol or other similar molecules to the TCE; however, these methods have been shown to decrease binding affinity [20,134]. HSA binding comes with the benefit of not effecting antigen binding affinity [20] and hence is the most common strategy to extend TCE half-life beyond Fc domains.

As previously noted, Fv-like TCEs have both light chains and heavy chains for binding to antigens. However, the light chains are not always required for binding to antigens, and sometimes, this binding can be achieved through heavy chains only. Antibodies consisting of only heavy chains can be found naturally and molecules consisting of only heavy chains can be artificially made for pharmacodynamic effect. These molecules are often called nanobodies or single variable domain on heavy chain antibodies [135]. These smaller molecules are starting to be considered for TCEs, where the TCE uses nanobodies for their binding sites. The aim of the nanobody-like TCE is to leverage the small size and unique properties of nanobodies in order to improve the therapeutic effect on solid tumors. The improved therapeutic effect comes from their small size, which allows for enhanced tumor penetration [136]. This has been observed in preclinical studies in which nanobody-based TCEs were able to increase intratumoral infiltration and hence reduce tumor burden in mice [137]. Nanobodies also have multimeric formation potential, which may allow the TCE to target more tumor antigens. This may allow the nanobody-based TCE to have higher tumor specificity and hence lower off-target toxicities compared to other TCE types [138]. These properties may allow for more effective and safer TCE therapies in solid tumors. However, as nanobody-based TCEs are still a novel treatment, more research will need to be done to properly verify these benefits. Furthermore, nanobody-based TCEs come with their own unique disadvantages that will need to be considered for clinical effect. As nanobodies are very small, they have a short serum half-life [135]. This may lead to the need for half-life extending modifications to the TCE to reduce the need for frequent dosing. It is also important to consider that nanobodies are more proficient at recognizing concave or conformation epitopes compared to flat or linear epitopes [135]. This may limit the nanobodies' range of epitopes that it can bind to, which may affect the efficacy of the treatment.

On-target, off-tumor toxicities is a severe problem limiting TCE therapy especially in solid tumors. In order to reduce these toxicities, prodrug TCEs are being designed. The idea behind prodrug TCE is that when initially introduced into the body, the TCE is unable to bind to its targets and activate a T-cell response [139]. Only once the TCE is close to cancerous cells is the TCE able to bind to cells and cause cytolysis. This effect is usually achieved through cleavable linkers attached to the TCE, blocking the binding sites. These linkers will cleave once under the right conditions, exposing the TCE binding sites. The linker is usually sensitive to enzymes found in high concentrations within the tumor microenvironment (TME), meaning most of the linker cleavage happens near the cancerous cells [140]. By having TCE only be able to activate a T-cell response in the TME, the options for which TAA to choose are increased as healthy cells also expressing the TAA have a decreased likelihood to be targeted by the TCE. This concept has been shown preclinically with mice showing little to no active TCE in healthy tissues, but showed active TCE in the TME when treated with a prodrug TCE [141]. The type of mask chosen may also have an effect on the TCE dynamics, peptide masks and protein masks may be linked to an HSA binding domain to improve the half-life of the TCE in prodrug form [140], and the size of the protein mask may intrinsically extend the half-life of the TCE [141]. By having the half-life extensions linked to the cleavable part of the TCE, when the drug becomes active and able to elicit a T-cell response, its half-life is reduced. This provides further protection against on-target, off-tumor toxicities, as if the active TCE diffuses away from the tumor, the short half-life means the TCE will be quickly cleared from the body, preventing harm to healthy tissues [141].

Beyond using peptide and protein masks, a new form of prodrugs is currently being developed with more complex mechanisms such as conditionally active COBRA (COnditional Bispecific Redirected Activation) TCE [140]. COBRA TCEs like MVC-101 (also known as TAK-186) work with a similar cleavable linker, but the mask is a protease-cleavable scFv linker connected to an inactivated variable domain, an HSA binding site. MVC-101 in its uncleaved form can bind to epidermal growth factor receptor (the chosen TAA) but cannot bind or interact with the T cells. However, unlike other masks, MVC-101 still cannot bind to T cells once cleaved. For MVC-101 to bind to T cells, it must first bind to the tumor cell and then create a dimer with another MVC-101 bound to the tumor cell in order for their anti-CD3 VH and VL domains to pair and create an active binding site for the T cells [142]. While the prodrug TCEs have great potential to reduce on-target, off-tumor toxicities, care is required to make sure the mask does not impact the efficacy of the treatment. If the mask is too insensitive to the TME, the TCE may not be able to elicit an effective response, while a mask that is too sensitive may cleave in healthy tissues and cause healthy tissue damage.

Another design aimed at preventing on-target, off-tumor toxicities is logic gated AND TCEs. Based on Boolean logic, these are a type of TCE that needs to bind to 2 tumor antigens in order to elicit a cytotoxic effect. This is typically achieved by designing the TCE to exhibit weak affinity for each individual tumor antigen, such that stable binding is only achieved through cooperative engagement of both antigens, thereby enabling sufficient time for TCE to promote synapse formation [143]. By choosing antigens that are coexpressed on cancer cells in high number, but have limited coexpression in other healthy cells, AND logic gated TCE can be more focused on cancer cells while avoiding healthy cells. This has been studied in both *in vitro* and *in vivo* models where healthy nontarget cells had minimal killing while the cancerous cells had increased killing [144,145]. Alternatively, instead of requiring both antigens to be bound to, some TCEs require only one antigen in order to elicit a cytotoxic effect, sometimes referred to as logic gated OR TCEs [143]. These OR TCEs aim to deal with tumor heterogeneity and antigen escape by giving the TCE more options to bind to, decreasing the likelihood that a cancer cell is resistant to the treatment. Furthermore, it has been observed that cancerous cells that display both antigen can have over a 1,000-fold greater potency compared to killing on cells displaying a singular receptor [146]. In a TCE that targets 3 antigens, a potential benefit of OR TCE may be reduced toxicities such as CRS as opposed to singular targeting TCE [147]. By increasing the number of antigens a TCE can attach to, it makes antigen escape of the cancer cells less likely. However, more receptor targets may increase the risk of TCE binding to healthy cells, causing on-target, off-tumor toxicities.

## Mathematical Models

During the development of TCEs from design to implementation, it is crucial to understand the biological mechanisms that drive both efficacy and adverse side effects. Mathematical models are an important component in understanding and predicting these mechanisms. Models can allow for powerful predictions such as optimum dosages, the effectiveness of drug designs, and the severity of adverse side effects and their management. With more immunotherapies being developed and data becoming available, the ability for mathematical models to accurately predict their outcomes is increasing. With reliable and precise models, the speed and effectiveness of drug development can be enhanced. However, there is still work to be done in improving the accuracy of models in this field. Hence, a review on current models in immunotherapy that relate to TCEs is useful to understand where the development of TCE-related models should go next.

As T cells are such an important cornerstone of many immunotherapy techniques, many models have been developed exploring T-cell mechanisms and interactions. The kinetic proofreading model and its development showcases this [148]. The kinetic proofreading model describes and simulates the initial signaling a TCR undergoes after binding to a peptide major histocompatibility complex (pMHC). The initial model is an algebraic and ODE-based model [149]. As stated in a review by Shi *et al.* [148], the initial model suggests that once a TCR has bound to a pMHC, it must go through intermediate steps before activation and can regress back at a constant rate to the initial state. The model suggested that $K_{A}$, defined as association rate divided by the dissociation rate, is responsible for the potency of a TCR against a pMHC. The model was further developed by other authors to include dynamics like faster rebinding after dissociation, coreceptor dynamics influencing the association parameters and hence affinity, and positive and negative feedback loops to capture phenomena missed in the previous model [148]. However, Shi *et al.* [148] highlight how the proofreading models do not account for costimulation or inhibitory molecules, showing the need to develop modules on TCR signaling further. More in-depth study of T-cell signaling may allow understanding of the consequences of skipping the binding to pMHC like in the TCE case.

Efforts have also been made to model T-cell exhaustion mechanisms. One such example is by Jansson *et al.* [150], which uses ODE compartment modeling to simulate the binding of B7-1 and B7-2 to CD28 and CTLA-4. Their model found that the binding affinity between CD28 and B7-1 and B7-2 was very sensitive and reducing the affinity would have a considerable impact on complex formation. However, CTLA-4 binding to B7-1 and B7-2 was insensitive and reducing this affinity by 10-fold had little impact on binding. The model also finds and agrees with the consensus that CTLA-4 outcompetes CD28 binding and, hence, is normally the dominant coreceptor when both are coexpressed, which is part of the mechanism in which CTLA-4 creates an inhibitory effect. The Jansson *et al.* [150] model has been expanded to include anti-CTLA-4 antibodies [151]. The model found that anti-CTLA-4 antibodies reduced the dominance of B7-2-bound CTLA-4 by around 75% after the “initial hours” (approximately 15 min into the simulation). Furthermore, the model found that anti-CTLA-4 antibodies had a minimal effect on CD28/B7-1 and CD28/B7-2 complexes. This case is important to highlight as it shows how models from T-cell behavior can be adapted to immunotherapies. As more complex TCE models are developed and combination therapies become more prevalent, combining models like this is increasingly more important and insightful to the field. Furthermore, the compartmental nature of the models lends itself well for expansion for new dynamics to modeled upon (such as the inclusion of TCEs).

### The Jiang model

When modeling TCEs, ODE compartment models are a prominent method used to simulate TCE dynamics. Many of the models focus on how TCE facilitates binding of T cells to cancer cells, and then relates a killing mechanism dependent on T cell to cancer cell binding. An example of this is that of Jiang *et al.* [152], which predicts TCE binding into 4 compartments. Initially, the drug is unbound and can bind to either a T cell or a cancerous cell, forming a dimer. Once bounded to one of the cells, the complex can either bind with the alternative cell not part of the complex, creating a T cell–drug–cancerous cell trimer, or unbind from the complex and become a free unbound drug again. Once bound into a trimer, there are unbinding rates to allow the trimer to become a T cell–drug dimer or a drug–cancerous cell dimer. This system of modeling TCE binding mechanics is not unique to Jiang *et al.* [152], and this style and its variations are often used to describe trimer mechanics. The model proceeds to average the number of TCE complexes per cancerous cell, and using this average defines a killing rate of the T cells using a Hill equation. T cells go through several delay compartments before contributing toward killing of the cancer cells. The evolution of the cancer cells is described by an exponential growth rate, subtracted by the killing rate multiplied by T-cell occupancy in the last of the delay compartments. The model also includes a control tumor, which has the same growth rate as the tumor being treated, but is unaffected by the TCE treatment. The control tumor is then compared to the treated tumor to calculate the cytotoxic effect the treatment is creating.

Compared to some other TCE models, the Jiang *et al.* model [152] has relatively few equations, allowing for easier understanding of model dynamics. This does not prevent the Jiang *et al.* model from being able to replicate *in vitro* data on different TCEs applied to differing cell lines. Using *in vitro* experiments, the model was able to replicate and predict the cytotoxicity of differing TCEs in different conditions such as TCE concentration, E:T ratio, and receptor expression levels. The sensitivity analysis of the model presents some interesting insights into TCE dynamics. An initial assumption one may have about TCE dynamics is that higher concentrations and binding affinity would result in more trimers being formed and, therefore, a higher tumor kill rate. However, the sensitivity analysis of Jiang *et al.* [152] challenges this assumption. In comparing differing TCE concentrations and binding affinity, the analysis finds that at lower values, the number of TCE complexes and the rate of tumor killing are low, as expected. As you increase the concentration and binding affinity, so does the killing rate and number of TCE complexes. This pattern continues until a sufficiently large concentration or affinity is reached; afterwards, any further increase of these values causes a reduction in cytotoxicity, due to the hook effect in trimer formation.

Models like Jiang *et al.*’s [152] are invaluable in understanding the relationship of dosage to efficacy; they allow understanding of the relationship of dosage to efficacy. This dose-to-efficacy relationship plays a major role to provide better insights into what concentrations are needed for an efficacious dosage *in vivo*.

It is important to note that Jiang *et al.*’s model [152] is an ODE model and comes with some fundamental assumptions. An ODE model inherently lacks a spatial component in each compartment; this may lead to ODE models missing out key mechanisms that the spatial element may contribute to. In a case like TCEs where a cancer cell and a T cell need to be close in order to form a synapse, the formation of this synapse would further affect spatial elements; leaving out these components may lead to models missing out key dynamics and mechanisms from TCE. When using the Jiang *et al.* model to predict TCE efficacy, it is important to also consider that many parameters depend on specific experimental scenarios, and the predictability of such models might be difficult to extend to other scenarios. While the model has great predictive ability, some of the parameters do not have a mechanistic grounding to them. If the model were to be expanded to shift toward a more mechanistic approach, this could allow for unique insights into TCE behavior to be learned and studied.

### The Flowers model

A similar model to Jiang *et al.* [152] is one proposed by Flowers *et al.* [153]. The model also focuses on the mechanisms of how TCE binds and creates synapses between T cells and cancer cells, with a similar compartmental binding structure as Jiang *et al.* [152], where the drug starts free and unbound and can either bind to a tumor cell or a T cell. After binding to one cell, the drug can bind to the other cell, creating the TCE trimer. Where Flowers *et al.* [153] differ from Jiang *et al.* [152] is the dynamics of cell–drug binding. In Jiang *et al.* [152], the binding rates were constant depending on the drug affinity, while in Flowers *et al.*, the binding dynamics are more dependent on the local environment. The dissociation rates going from trimer to dimer and dimer to free are constant; however, the association rates going from free to dimer and dimer to trimer depend on which stage of binding the drug is in. In the free-to-dimer stage, the association rate is the constant binding rate divided by the reaction volume. Flowers’ model aimed to create a new model for more accurate TCE modeling; hence, for the second step (dimer to trimer), Flowers *et al.* [153] proposed 2 models: the “3D” model and the “2D” model. The “3D” model is made to represent previous models’ “well-stirred” approach [154,155] and so the dimer-to-trimer step is similar to the free-to-dimer step, with a factor $\chi_{3D}$ multiplying the on-rate of the second binding step to account for avidity. The aim of the Flowers *et al.* model [153] is to incorporate trans-cell cross-linking to the model instead of an avidity approach, called the “2D” factor. This is achieved in the second step by replacing $\chi_{3D}$ with a $\chi_{2D}$ that depends on the sum of the surface areas per effector cell and target cell. The reason for the naming comes from the derivation of the model, where the model assumes the binding step takes place on a 2D surface area and from this assumption derives the second binding step previously described. For both models, the receptor occupancies are then calculated for both T cells and the tumor cells using the models’ output.

Flowers *et al.* [153] compared the “3D” model to their derived “2D” model. Both models had a similar hook effect on receptor occupancy that was seen in Jiang *et al.* [152], with the χ factor also being a parameter that followed the hook effect. When comparing the 2 models to both *in vivo* and *in vitro* data, Flowers *et al.* [153] found that the “2D” model had more realistic and accurate predictions than the “3D” model, suggesting that models that incorporate trans-cell cross-linking may be more accurate than the generalized “well-stirred” assumption. However, both models are able to accurately predict the data. Flowers *et al.* [153] suggest that the strength of the “2D” model is found in the sensitivity analysis. The “3D” model is more sensitive to cell density than the “2D” model, which allows the “2D” model to be more consistent to data in varying circumstances compared to the “3D” model.

Like Jiang *et al.*’s model [152], Flowers *et al.*’s model [153] is an ODE model, which means it also lacks a spatial element. Expanding both models to include this spatial element could gather unique insights, especially to see if a spatial element affects the “2D” and “3D” in similar or differing ways. This comparison could garner unique insights on both the model and TCE dynamics. Flowers *et al.* [153] also suggest ways the model could be expanded, such as adding more compartments to describe the TCE distribution in the tissue and other components of the body, pushing the model more into physiologically based pharmacokinetic modeling (PBPK). Another possible expansion suggested is to a relation of trimer formation to T-cell activation, leading to cell killing and cytokine release. This expansion could be very insightful to TCE behavior; however, it is important that this is done with care as the addition of more parameters leads to a risk of reduced parameter identifiability, which may lead to some parameters to be difficult to estimate experimentally.

### The Ma model

The Jiang *et al.* [152] and Flowers *et al.* [153] models focused on the binding mechanics of the TCE, describing different mechanics when the TCE is sufficiently close for tumor cells to bind. Other models focus more on the pharmacokinetics and pharmacodynamics of the TCE in the body, such as the model described by Ma *et al.* [155], whose model has been reiterated and adapted upon to include differing TCE mechanics in other models. The model is an ODE-based model, which is adapted from a model that simulates the effect of anti-PD-1 on non-small cell lung cancer developed by Jafarnejad *et al.* [156].

The model describes differing compartments of the body, specifically the blood, the tumor, tumor draining lymph nodes, and peripheral organs. The model incorporates the dynamics of antigen-presenting cells trafficking to the lymph nodes, activation of the differing types of T cells, and T-cell dynamics including exhaustion. Trimer formation between target antigen and T cells is included as in previous models. However, Ma *et al.*’s model [155] was used to investigate cibistamab, which is bivalent for carcinoembryonic antigen (CEA). Therefore, the model also distinguishes between both of the target antigen binding sites, allowing for the case of both or only one of the target antigen sites to be bound to the CEA. The model exhibits a hook effect in TCE concentration. The model also found that TCE trimer formation with bivalent CEA binding was sensitive to the intrinsic cross-arm binding efficiency λ and the CEA density. Interestingly, if intrinsic cross binding efficiency was high enough, the bivalent TCE trimer formation was insensitive to CEA density and vice versa. When simulating a cohort of virtual patients, the model found that response was highly dependent on tumor mutational burden and effector T cell/regulatory T cell ratio, suggesting that TCE therapy may be heavily dependent on the condition and dynamics of the patient’s tumor and TME.

The model was then expanded to include a combination therapy between TCE and anti-PD-L1 inhibitors [118]. The model was used to show that patients who did not have a complete response from monotherapy were able to benefit from combination therapy. The model suggests that similar parameters that determined efficacy of TCE monotherapy also contributed to the potential success of the combination therapy. Anbari *et al.* [157] further developed the T-cell dynamics of the model by including helper T cells, and using multidimensional synergy of combinations (MuSyC) to give a more quantitative analysis of the synergy of the therapies. The MuSyC analysis found that the therapies had small syngenetic efficacy and potency. The authors suggest that this may be caused by the therapies having independent mechanisms of action to each other.

Models that simulate differing compartments are invaluable in showing the body-wide effect TCEs have. This allows biologists to understand the interplay on how TCE affects the body, and how the different conditions of the body affect TCE efficacy. Furthermore, the expansion to combination therapy is invaluable in helping to understand in which scenario are patients likely or unlikely to benefit from combination therapy, allowing for more careful and considered application.

The model by Ma *et al.* [155] consists of 68 ODEs and 105 algebraic equations as reported by the authors. A model of this size comes with many strengths; it can show a potential very detailed description of how TCE may interact in the situation modeled. It is also very useful in testing different scenarios and learning how interdependent certain dynamics are and how they affect each other and the entire system. However, a model with so many variables and parameters can come into issues of parameter estimation in experimental studies and clinical trials. Furthermore, with so many parameters, it is likely that many sets of parameters estimate similar results, which could make the confidence you can have in a particular parameter set weaker. This confidence may also then lead to a reduced confidence in the accuracy of the model’s ability to test different scenarios. The authors themselves admit some model dynamics had to be either assumed or ignored during model creation, suggesting that further validation of the assumptions is needed.

### The Abrams model

Bispecific TCEs are not the only type of TCE currently being modeled. Abrams *et al.* [158] demonstrate this with their trispecific TCE treating multiple myeloma model. The trispecific TCE being modeled has 3 binding sites that target CD38 on cancerous cells, CD3 on T cells, and CD28 on T cells, which enables binding to both tumors and T cells. The purpose of the CD28 binding is to costimulate naive T cells while also being expressed on tumor cells, enabling for the TCE to enhance the T-cell response while also having additional target antigens to bind to on the cancerous cells. The model focuses on T-cell mechanics, including naive, effector memory, and active T cells of differing lineages. As the model can include ineffective synapses such as inactive T cell and tumor synapses, the model also takes into account which cells the TCE are bound to and models an appropriate kill rate (if any) depending on the context.

When performing sensitivity analysis on the model, Abrams *et al.* [158] found that low-dosage CD3 binding affinity was a key factor in facilitating cancer killing as well as CD28. The higher affinity to T cells allowed for more T-cell activation, which is necessary for the immune system to engage an effective response. However, at higher dosages, they became less important, and a high CD28 binding rate could lead to ineffective synapses being formed. At lower dosages, a higher CD38 off rate was shown to decrease ineffective synapse formation as the cancerous cell dissociating from the TCE could bind to a new potentially effective synapse instead; however, this benefit was reduced at higher dosages as most cells were already engaged. Abrams *et al.* [158] also analyzed the model by comparing the normal trispecific TCE to a variant without the CD28 binding arm, to see if the trispecific nature of the TCE provided any benefits. They found that the variant followed a hook effect of a standard bispecific TCE. The trispecific case follows a different trend: as the dosage increased, the number of receptors occupied also increased as expected. However, once reaching its peak, the number of receptors engaged may slightly decrease, but overall, the number plateaus near the maximum number or receptors engaged. This suggests that, in some scenarios, trispecific TCE may not suffer as readily from reduced efficacy at higher dosages, unlike bispecific TCE.

The Abrams *et al.* model [158] is also an ODE model, which the authors report have 760 species and 114 parameters. This size magnifies both the strengths and weaknesses of the qualities big models come with that we previously discussed. Comparing Abrams *et al.* to previous models shows an occurring trend in the literature that many TCE models lean toward ODE models with large numbers of parameters, which often results in these models lacking a mechanistic foundation. While it is evident that these models bring great insights into TCE behavior, more models that are smaller and have more spatial elements may lead to interesting new discoveries on TCE behavior and could explain how some under-represented TCE mechanisms work, while improving their clinical applications and ability to explore scenarios, which are otherwise difficult to explore experimentally.

### The Liu model

While the previous models give valuable insight into the dynamics of TCE, they lack a spatial component in their considerations. To achieve a spatial element, agent-based models have been developed to study these dynamics in TCE. An example of this is a model of synapse formation by Liu *et al.* [159]. The authors used an agent-based approach to binding mechanics, including heterogeneity in tumor cells to stimulate tumor evolution.

For an immune synapse to be created, the cells must go through 2 steps, cell–cell encounter and cell–cell adhesion. The model simulates stationary target cells and effector cells moving independently in Brownian motion. The probability of cell–cell encounter is dynamic and depends on differing tumor microenvironment characteristics such as E:T ratio and cell density, with number of free target cells being the characteristic with the largest impact on cell–cell encounter. When an effector cell makes physical contact with a tumor cell, the model moves to the cell–cell adhesion stage where the effector cell may bind and adhere to the tumor cell or diffuse away. The probability of cell–cell adhesion is dependent on the area of contact between the cells and trimer formation. Trimer formation is assumed to occur in 2 steps, the first being dimer formation. Dimer formation follows similar mechanics to models such as Jiang *et al.* [152], where the TCE can bind and unbind from both CD3 on effector cells and the TAA on the target cells. This step in which the model calls the “3-dimensional binding reactions” is assumed to occur much faster than the rest of the dynamics of the model in the period the model is simulating, allowing for a rapid equilibrium assumption of the dynamics. The rapid equilibrium assumptions remove the requirement to simulate 3D binding reactions with ODEs and instead may be calculated using algebraic equations. Once cell–cell encounter has occurred, the model simulates the 2D binding reaction. The 2D binding reaction again follows a similar style to Jiang *et al.*’s [152] dimer-to-trimer formation. However, Liu *et al.* [159] include 2 intermediate terms, 1 term to account for antigen encounter and 1 term to account for antigen rotation. Once these intermediate terms or “virtual complexes” [160] have been created, the system then allows for trimer formation through chemical binding. These intermediate terms also include dissociation; i.e., the trimer can dissociate into a virtual complex and the virtual complex can dissociate into dimer and CD3/TAA.

Once adhered, the effector cell will create an immunological synapse leading to the eventual death of the tumor cell. Liu *et al.* [159] simulated “immunological synapse variants”, in which multiple effector cells attach to one tumor cell or multiple tumor cells attach to one effector cell. In terms of tumor elimination, however, immunological synapse variants eliminate the affected tumor cells with the same mechanics as the standard tumor cell–effector cell immunological synapse. Liu *et al.* [159] use the agent-based nature of the model to study the heterogeneous expression of CD19 (the tumor target antigen) on the tumor cells and CD3 on effector cells, with mechanisms to slowly down-regulate the expression of these antigens from internalization. Liu *et al.* [159] considered differing body compartments such as the blood and gut, in order to stimulate the differing dynamics of TCE in these locations.

The simulation and analysis of this model include results that support findings in previous models in addition to new unique findings the agent-based nature of the model allows. The hook effect discussed previously was predicted and replicated, including the same rationale on its occurrence. The model can accurately predict the number and type of immunological synapse variants formed during therapy. The authors showed that high E:T ratios promote the creation of effector cell–target cell–effector cell synapses, while lower effector cell:tumor cell ratios promote target cell–effector cell–target cell synapses. While the model did not consider the different effects immunological synapse variants may have on therapy dynamics, in clinical practice, these variants may have an effect on T-cell dynamics (such as T-cell exhaustion and tumor kill rate) and hence should be considered in the design of TCEs. As previously discussed, cancer evolution through antigen escape is a leading factor of TCE resistance, and the model supports this theory. The model was able to show that the tumor evolved toward lower-expressing CD19 cells and that lower expression of CD19 on cells reduced the chance of effector cells engaging an immunological synapse with them. The evolution of the cancer was not linear; rather, its rate increased over time. Furthermore, factors such as TCE concentration and immune synapse formation promoted faster and more extensive evolution, respectively. Evolution of the cancer may also be affected by tumor location. The model was used to find that the higher number of effector cells in the lymph nodes and spleen allowed for greater cancerous cell killing compared to other compartments which suffered from lower effector cell count. The model predicted that these sites of lower effector cell count allow the cancer to survive and adapt to the treatment, becoming what the authors refer to as “sanctuary sites” for the cancer. Even in cases where the model predicted 99% of the cancerous cells eliminated, organs like the bone marrow held a small number of low-expressing CD19 cells, which eventually repopulated the killed cancerous cells and caused patient relapse.

An agent-based model focused on synapse formation like Li *et al.*’s [161] gives valuable insight into synapse mechanisms. It can inform biologists under which conditions are the different types of synapse likely to form, which may be useful if biologists have a preference to a certain type depending on the TCE. Furthermore, the “sanctuary sites” that cause patient relapse are vitally important to understand and help guide future development and research to tackle and minimize these situations. It can also be used to help screen which patients are at highest risk to relapse so that earlier action can be taken to improve patient prognosis.

As an agent-based model, Li *et al.*’s model [161] brings unique insights into TCE dynamics. However, some simplifications were made in order to achieve this when compared to more traditional ODE models. For example, the model assumes that when a cancer cell and a T cell form a synapse, the cancer cell dies in the next time step. This behavior may be an oversimplification of TCE dynamics; as seen in previous models, there is a strong evidence to suggest that a larger trimer formation may lead to a stronger cytotoxic effect. This is explored in more detail however in Liao *et al.*’s model [160]. It is also important to note that encounter chance of cells is probabilistic by nature, meaning the model also lacks a spatial element. A potential expansion to make this model spatial could lead to unique insights into the causes of immunological synapse variants; this, in turn, would also allow for greater visualization of the model dynamics and outputs.

### The Liao model

Using the framework developed by Liu *et al.* [159], Liao *et al.* [160] expanded the model to focus more on replicating data. The model has a very similar structure to Liu *et al.* [160], with minor adjustments to some terms (such as the 2 intermediate complexes being combined into one as part of the 2D binding step). The main expansion the model focuses on is more in-depth mechanics on how the TCE elicits a cytotoxic effect after synapse formation.

Using the probability of cell–cell encounter and cell–cell adhesion, the model calculates the change in immunological synapses. The model assumes that the cytotoxic effect of the TCE follows a Hill equation, dependent on the percentage of tumor cells engaged in an immunological synapse, with a baseline cytotoxic effect when no immunological synapses are formed (referred to as the synapse–efficacy relationship). The model also separately calculates the cytotoxic effect using a similar Hill equation but instead using the concentration of free TCE (referred to as the concentration–efficacy relationship). The Hill equation for the concentration–efficacy relationship relies upon the classical *E*_max_ (concentration at maximal efficacy) and EC_50_ (concentration at half-maximal efficacy) in calculating the cytotoxic effect. Alternatively, for the synapse–efficacy relationship, the Hill equation relies on ES_max_ (synapse percentage at maximal efficacy) and ES_50_ (synapse percentage at half-maximal efficacy).

The model was accurately able to predict and simulate results from clinical trials of differing TCEs such as blinatumomab and teclistamab using ES_max_ and ES_50_ values that were derived from preclinical data. Liao *et al.* [160] also derived the *E*_max_ and EC_50_ values from preclinical data, but found these values to be highly variable to experimental specific data such as E:T ratios, binding affinities, and cell lines. In contrast, Liao *et al.* [160] found that ES_max_ and ES_50_ were less volatile to the experimental specific data and remain consistent through changes of factors like E:T ratios and binding affinities. After simulation, sensitivity analysis on binding affinity was performed on the model and the hook effect was shown, that is, less synapse will form at higher affinities. From plotting the sensitivity analysis, Liao *et al.* [160] show the affinities of both mosunetuzumab and especially blinatumomab to be higher than the optimal value for synapse formation. However, Liao *et al.* [160] note that other factors such as dosage affect cytotoxicity, suggesting a nonlinear relationship between synapse formation and cytotoxicity.

As a model built upon Li *et al.*’s [161], many of the pervious suggestions discussed apply to Liao *et al.*’s [160] model. However, the authors bring up their own suggestions on potential future expansions to the models. The authors note the importance of dynamics like CRS, T-cell activation, and T-cell expansion in understanding TCE effectiveness and toxicity, suggesting that expanding their model to include these dynamics is vital in order to fully understand TCEs. The authors further note that these dynamics are essential to understand how longer-term TCE efficacy affects patients, as relapse probability relies heavily on T-cell dynamics, noting that this may limit the model’s ability to predict longer-term patient dynamics.

### The Su model

The models discussed previously focused more on the cellular level; however, focusing on the intercellular level may provide useful results on TCE dynamics. Su *et al.* [71] created an agent-based model in order to analyze the intercellular scale of TCE binding alongside the cellular scale. At the intercellular level, the model describes binding of TCE to CD3 on T cells and TAA on normal cells. The antigens are distributed randomly on the corresponding cell’s surface, and the TCEs are distributed within the volume between the 2 surfaces. The intercellular dynamics are modeled using a kinetic Monte Carlo algorithm to model both receptor and antibody movement as well as their binding interactions. The model also incorporates the structure of TCE, such as linker length, to limit how far a binding arm can reach. The model simulates T cells, tumor cells, and normal cells randomly distributed in a spherical rigid body, using Langevin dynamics to calculate cell movements and interactions. Unlike previous models, Su *et al.* incorporate deeper cell dynamics such as friction from the environment and other cells, repulsive energy between cells, and small deformations between contacting cells. The simulation from the intercellular model is used to determine adhesion strength in the multicellular model.

When analyzing this model, the hook effect is seen in the intercellular simulation. As the number of antibodies increases, the number of intercellular bonds follows the hook effect, with the same reasoning of binary complexes saturating the receptors. The shape of the hook effect depended on different factors in the system; more receptors allowed the peak number of intercellular bonds to increase while also requiring more TCEs to reach the saturating effect. Binding affinity also determined the shape of the hook effect: high affinity caused the saturating effect to occur with a lower amount of TCE, while an intermediate affinity still reached the same peak, but at a higher number of TCEs in the system. A low affinity caused a lower peak of bonds and had overall worse performance than intermediate affinity. The model also simulates different linker lengths to see the impact on trimer formation. The model found that around 2.5 nm was an optimal length for the linker for creating intercellular bonds. A linker shorter than 2.5 nm had less searching space to find the other receptor, while a linker longer than 2.5 nm had too many degrees of orientational freedom, making binding to the receptor more difficult.

The model by Su *et al.* [71] provides an excellent framework to understand how TCE design may affect intercellular dynamics. Models like Su *et al.*’s [71] may be used to inform the preclinical design of TCE, helping biologists design optimal linker lengths and affinities. The authors note that this is a proof-of-concept study and will include further expansions to the dynamics, such as plasma membrane undulations, a more heterogeneous receptor cell distribution, and linker flexibility. These improvements may allow for further fine-tuning of TCE design and equip biologists with greater knowledge on the benefits and drawbacks of TCE designs. A further expansion to this model could be linking the impact of these trimers on costimulation and T-cell activation, to understand how the intercellular dynamics link to the overall TCE dynamics. A computational link may further empower biologists to further understand how their TCE designs link directly to TCE effectiveness and toxicity.

## Conclusions

TCEs are complex therapies with many intertwined and dynamic components, which creates challenges in their implementations and predictions. From both the design phase and application phase, there are many challenges that need to be addressed in order to fully realize the potential of TCEs. With the recent increase in TCE approvals and the development of novel designs aimed at overcoming current limitations, the potential of TCEs as an effective cancer immunotherapy continues to grow. As the field of TCEs expands and develops, new challenges and questions emerge, in which mathematical models are in a prime position to help develop and understand the TCE field. As discussed, current models are able to predict and give valuable insights into the dynamics of TCEs. Current models can show a wide array of results such as the hook effect, the effect trispecific binding has on TCE efficacy, and how TCE design can affect trimer formation on an intercellular level. These models help develop the theory of TCE and can help guide TCE in both the design and clinical setting.

It is important to consider that this review is not comprehensive of every TCE model out there. During this review, we have focused on more mechanistic and PBPK models; however, it is important to note that there are other strategies used to model TCEs. Machine learning or hybrid modeling approaches that combine mechanistic and data-driven methods are becoming more prevalent in biomathematics. These approaches have the power to aid in the TCE design stage, allowing for ML-guided TCE structure design and deep learning for antigen selection. Furthermore, although this review focused on models specifically designed to simulate TCE activity, more generalized modeling approaches may also prove valuable in advancing our understanding of TCE-mediated responses. PBPK models on bispecific antibodies that focus on tissue distribution, lymphatic recirculation, convective transport, and FcRn recycling may give valuable insights into how TCEs transport throughout the body.

In this review, we have considered both the mathematical and biological perspectives of TCE separately, each field highlighting various problems and challenges in studying TCEs. However, taking a step back and considering both fields together gives us a unique perspective that only a dual review like this one can provide. When reviewing the mathematical models, there is a clear trend toward focusing on binding mechanisms, and many papers focus on what mechanisms are required to enable TCE binding and how does that result in cell killing [71,152,153,159,160]. Each paper has its own unique approach to this question and is able to approximate clinical data. There are some models such as that of Ma *et al.* [155] that take a wider approach, focusing on modeling more broad immune system dynamics and combination therapies.

Typically, however, a large majority of papers focus on binding dynamics without considering the wider nonbinding dynamics, allowing for interesting and novel binding-related mechanisms to be studied that are difficult to study clinically or experimentally, such as the hook effect. Binding dynamics, however, cannot account for all issues currently facing TCE efficacy; many factors such as immune escape, CRS, T-cell exhaustion, and solid tumor characteristics are large obstacles that need to be addressed. With the modeling focus being so high on binding mechanisms, the other factors that influence TCE efficacy are under-represented in the modeling literature. This further extends to the techniques being developed by biologists to tackle these issues such as step-up dosage and masking. Incorporating the various biological mechanisms beyond binding dynamics like deeper immune system mechanisms and solid tumor characteristics into mathematical modeling frameworks could potentially help to explain some behaviors that may be difficult to study experimentally, in a manner similar to how the hook effect is currently being studied. Furthermore, these factors may have an unforeseen influence on currently developed binding models; hence, by including these under-represented dynamics, further insights may be found for currently modeled phenomena like the hook effect. Through this review, we aim to highlight the importance for mathematical modelers to stay informed in current developments in the TCE field and to create new models for these developments, allowing for further understanding of novel biological mechanisms and strengthening of previous mathematical findings.

A common trend that repeatability occurred from mathematical modeling is the hook effect. Many models predict that the hook effect is a phenomenon in TCE, yet as previously discussed, it is not focused on clinical settings, with arguments that the current toxicities are a limiting factor preventing the high concentrations required for the hook effect [70]. However, in the mathematical models, a “top down” approach is taken, focusing on the overall population dynamics. This top down approach with a lack of spatial modeling presents an important question. How does the spatial element affect the hook effect on a more local area? In many cases, the TCE is not uniformly distributed in the body, with some cancer cells and T cells binding to more TCE than others. Could this lead to a hook effect only on a small population of cells? Is it possible that certain local regions of cells accumulate TCE concentrations high enough to induce the hook effect, reducing the cytotoxic efficacy of the T cells and creating the "sanctuary sites" described by Li *et al.*'s [161] model? If this is the case, is this a potential cause of patient relapse in cancer treatment? With so many potential unknowns about the clinical application of the hook effect, the need to evolve current mathematical models to include more individualized local and spatial elements to study this phenomenon is imperative.

As discussed, there is room for growth in the field of TCE mathematical models, and such models may address several unanswered questions. More specifically, are there better dosing strategies than the ones in common use? How much impact does the hook effect have on TCE clinical outcomes? Does that impact change over time? Current models may benefit from a deeper analysis of the hook effect to gain a deeper knowledge of its mechanisms and effects on therapy, which, in turn, may guide the development of safer and more effective clinical trials. The TCE field may also benefit from more spatial modeling of solid tumors, with dynamics of TCE structure affecting tumor interaction, such as penetration and linker length. Future models may also benefit from keeping all parameters mechanistic and identifiable, in order to allow easier application and predictions for future TCE and to give more meaning to sensitivity analysis. Mechanistic models that focus on TCE structure may help biologists gain a deeper understanding of what makes TCE design efficacious and safe, promoting construction of new TCE designs that leverage this new knowledge. In addition to current models, the field may benefit from the study and modeling of less explored dynamics of TCEs.

Step-up dosage and its relation to CRS still has many unanswered questions that may benefit from deeper analysis, such as which dosages should be used for step up in order to maximize the efficacy of the treatment and the reduction in CRS, and whether other unimplemented dosage techniques may be more advantageous. With CRS being such a large issue in TCE therapy, the need for more mechanistic models that predict T-cell mechanisms, including T-cell activation and cytokine release dynamics, is only increasing. Well-calibrated CRS models sensitive to patient parameters may allow for greater management and prevention of severe CRS. This would then allow deeper optimization of dosage to allow for a higher, safer cancer cell killing.

One of the biggest issues facing TCE previously discussed is the reduction of effectiveness over time, through both T-cell exhaustion and antigen escape. Many models currently fail to capture these mechanisms, limiting the TCE model’s ability to accurately predict long-term patient prognosis. Developing more models that explore T-cell exhaustion during TCE therapy may allow for unique insights into mechanisms that cause T-cell exhaustion, which patients are at risk of a higher rate of T-cell exhaustion, and potential remedies that clinicians could employ to reduce T-cell exhaustion. Similarly, more modeling of escape mechanisms could also allow greater insights into whether certain antigens are more prone to antigen escape than others, and the effect dosage has on antigen escape.

Models have already guided dosage in previous clinical trials [36,90], and so answering these questions with modeling may give guidance and confidence to clinicians in their future dosage regimens. Development on models that focus on virtual patients may allow for more in-depth patient stratification, allowing for individualized dosage optimization and risk management to greatly improve patient prognosis. Models that include more dynamics based on the structure of the TCE may also allow for greater comparison of differing TCEs. Dynamics of tumor penetrability, linker length, half-life, and binding affinity all differ between different TCEs, and having more models include these dynamics will allow for deeper comparisons of current TCE structures. This, in turn, will aid in more informed decisions for the development of future TCEs.

## Data Availability

This is a review article and generated no new data. All data underlying this study are cited in the references.
